# Proximal Tibial Epiphyseal Injury in a 14‐Year‐Old Asian Male With Vitamin D Deficiency as a Possible Cause: A Case Report

**DOI:** 10.1155/cro/9556018

**Published:** 2026-04-23

**Authors:** Shotaro Kawamura, Kenta Kamo, Hidehiko Kido, Akihisa Haraguchi, Yoshihide Shinjo, Shigemasa Kuga

**Affiliations:** ^1^ Department of Orthopaedic Surgery, Yamaguchi Red Cross Hospital, Yamaguchi, Japan; ^2^ Department of Orthopaedic Surgery, Graduate School of Medical Sciences, Kyushu University, Fukuoka, Japan, kyushu-u.ac.jp

**Keywords:** adolescents, case report, pediatric fracture, proximal tibial epiphysis, Salter–Harris fracture, vitamin D deficiency

## Abstract

We report a case of a 14‐year‐old Asian male who presented with a Salter–Harris Type II injury of the proximal tibial epiphysis and a fibular fracture following a minor mechanism of injury. The patient was emergency transported to our hospital with complaints of pain and flexion deformity around the left knee following a contusion. Imaging studies confirmed the diagnosis of a Salter–Harris Type II injury of the proximal tibial epiphysis. Given the minor mechanism of injury, blood tests revealed vitamin D deficiency. Under general anesthesia, manual reduction and percutaneous pin fixation were performed, resulting in good bone union and functional recovery. This case highlights the importance of considering vitamin D status when managing pediatric fractures following minor mechanisms of injury or unusual fractures and epiphyseal injuries.

## 1. Introduction

Proximal tibial physeal injuries are uncommon, accounting for approximately 0.6%–2.1% of all physeal injuries [1, 2] and primarily occur during early childhood, typically between the ages of 3 and 6 [3]. These injuries are often reported to result from falls or collisions involving relatively high energy [4]. It is uncommon for such an injury to occur at the age of 14 with a minor mechanism of injury, as in this case. In this paper, a minor mechanism of injury is defined as an injury caused by less energy than that generated by falling from a standing position, in accordance with the WHO definition [5]. Additionally, vitamin D deficiency has recently gained attention as a risk factor for pediatric fractures. Blood vitamin D levels below 20 ng/mL have been reported to increase the risk of fractures [6]. This is because vitamin D is essential for bone mineralization and contributes to maintaining normal growth plate function [7]. Furthermore, it has been suggested that certain diseases causing vitamin D deficiency may render the growth plate cartilage vulnerable, potentially facilitating epiphyseal injuries [8]. In previous case reports of proximal tibial epiphyseal fractures, vitamin D deficiency has been suspected as a contributing factor [9]. In this report, we present an uncommon case of a male adolescent patient with a proximal tibial epiphyseal fracture and Salter–Harris Type II fracture caused by a minor mechanism of injury, in whom vitamin D deficiency was identified.

## 2. Case Presentation

A 14‐year‐old Asian male tripped and fell forward while cleaning at school, striking his left knee in a flexed position against the stairs, resulting in injury. Immediately after the injury, he experienced pain in the left knee and a flexion deformity of the proximal tibia, with the left knee unable to extend and difficulty walking, leading to emergency transport to our hospital.

### 2.1. Physical Examination Results

There were no significant past medical conditions other than constipation, which had been treated at a local clinic 2 weeks prior to the injury. The patient and his parents confirmed that there was no history of easy fractures, food allergies, or picky eating. The patient′s height was 173 cm (+1.1 SD), weight was 63.8 kg (+1.3 SD), and BMI was 21.3, with no obesity or malnutrition.

At the time of examination in our department, the patient′s vital signs were stable. There was a contusion around the left knee, but no open fracture was observed. The left knee was flexed at 100° and could not be extended. The popliteal artery and dorsalis pedis artery were palpable, and the ankle and toes had active movement. No neurological abnormalities such as numbness, sensory abnormalities, or muscle weakness were noted.

### 2.2. Results of Pathological Tests and Other Investigations

**Figure 1:**
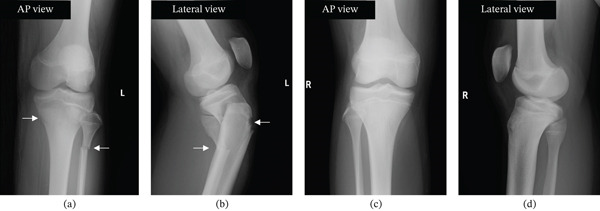
Initial radiographs of both knees. (a) Plain x‐ray image of the left knee joint at the time of the initial visit to our department. Anteroposterior (AP) view of the left knee showing a proximal tibial physeal fracture (arrow) and a fibular fracture (arrow). (b) Plain x‐ray image of the left knee joint at the time of the initial visit to our department. Lateral view of the left knee demonstrating displacement of the proximal tibial physeal fragment (arrow) and a fibular fracture (arrow). (c, d) Plain x‐ray image of the right knee joint at the initial visit to our department. There are no findings suggestive of rickets, such as cupping or flaring: (c) AP view and (d) lateral view.

**Figure 2:**
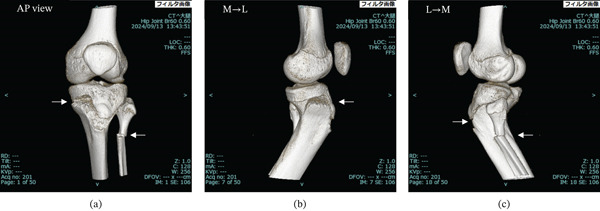
Initial plain CT images of the left knee. (a) Plain CT image of the left knee joint at the initial visit to our department. There is a Salter–Harris Type II fracture (arrow) of the left proximal tibial epiphyseal line and a fracture (arrow) of the proximal fibular shaft. (b) Plain CT image of the left knee joint at the initial visit to our department, medial to lateral view. There is a Salter–Harris Type II fracture (arrow) of the left proximal tibial epiphyseal line. (c) Plain CT image of the left knee joint at the initial visit to our department, lateral to medial view. There is a Salter–Harris Type II fracture (arrow) of the left proximal tibial epiphyseal line and a fracture (arrow) of the proximal fibular shaft.

Based on plain x‐ray and plain CT imaging, the diagnosis was a Salter–Harris Type II injury of the proximal tibial epiphysis and left fibular fracture (Figures 1a,b and 2).

**Figure 3:**
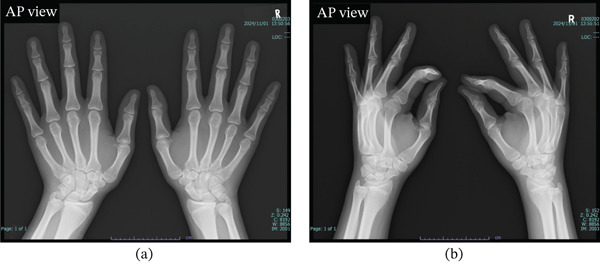
Initial radiographs of both wrists. (a, b) Plain x‐ray images of both wrist joints at the initial visit to our department. No findings suggestive of rickets, such as cupping or flaring, were observed (AP view).

Given the minor mechanism of injury and the rarity of the epiphyseal injury, rickets and vitamin D deficiency were considered differential diagnoses, and additional tests were performed, including plain x‐ray images of the wrist and blood tests to evaluate serum calcium, serum phosphorus, serum alkaline phosphatase (ALP), and 25‐hydroxyvitamin D (25OHVitD) levels. Plain radiographs of both wrists showed no findings suggestive of rickets (Figures 1c,d and 3).

Blood tests revealed serum calcium levels of 9.4 mg/dL, serum total phosphorus levels of 3.5 mg/dL, serum ALP levels of 226 IU/L, and serum 25(OH) vitamin D levels of 15 ng/mL. Calcium, phosphorus, and ALP were within the normal range (Table 1). Serum 25(OH) vitamin D levels were deficient [10].

**Table 1:** Patient′s preoperative laboratory data.

Lab	Result	Normal range
Calcium	9.4 mg/dL	8.6~10.1 mg/dL
Phosphorus	3.5 mg/dL	2.4~4.6 mg/dL
ALP	226 IU/L	122.5~472.5 IU/L
25‐Hydroxyvitamin D [25(OH)D]	15 ng/mL	≧ 30 ng/mL: Sufficient 20–29 ng/mL: Insufficiency < 20 ng/mL: Deficiency

### 2.3. Differential Diagnosis

This is an uncommon case of epiphyseal injury in an adolescent boy due to minor trauma, with a suspected fragility fracture. The differential diagnoses included diseases that cause bone fragility, such as rickets and hypophosphatasia. However, based on the plain x‐ray images and blood test results, rickets and hypophosphatasia were ruled out, and only vitamin D deficiency was identified.

### 2.4. Treatment Plan

**Figure 4:**
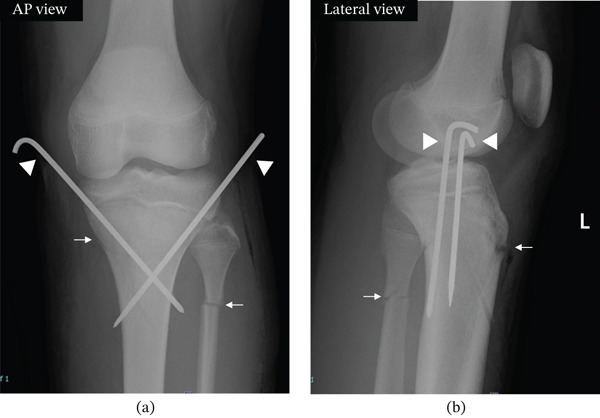
Postoperative plain x‐ray images of the left knee. (a) Plain x‐ray image of the left knee joint immediately after surgery. The tibial fracture line (arrow) and fibular fracture line (arrow) were also visible. Two 3.0‐mm K‐wires (arrowheads) were inserted bilaterally into the medial and lateral aspects of the proximal tibial fragment using a cross‐pinning technique. Intraoperative findings showed improved stability. Additionally, following reduction and fixation of the left tibia, the left fibula achieved an appropriate reduction position. The left fibula had stable contact and no instability, so fixation was not performed (AP view). (b) Lateral view of the left knee joint on plain x‐ray immediately after surgery. The tibial fracture line (arrow) and fibular fracture line (arrow) were also visible. Two 3.0‐mm K‐wires (arrowheads) were inserted bilaterally into the medial and lateral aspects of the proximal tibial fragment using a cross‐pinning technique (lateral view).

It was decided to perform surgical treatment on the day of admission. Under general anesthesia, traction was applied to the left lower leg, and the anterior aspect of the left knee joint (particularly around the tibial tuberosity) was compressed from the front. This relieved the flexion deformity and achieved an appropriate reduction. However, upon releasing the traction and compression, the flexion displacement recurred. Furthermore, during manual flexion and extension of the knee joint for the purpose of ROM assessment, the flexion deformity readily reappeared, with marked instability evident. Consequently, we determined that manual reduction alone could not maintain the reduced position, rendering conservative treatment inadequate. Due to residual instability, a 3.0‐mm K‐wire was inserted at the medial and lateral midpoints of the left proximal tibial fragment, angled 45° laterally from the tibial axis, and advanced until it penetrated the contralateral cortical bone, thereby creating a cross‐pinning configuration to stabilize the proximal and distal fragments. The intersection of the cross‐pinning was positioned distal to the fracture line. After K‐wire insertion, the stability of the fracture site was assessed. Flexion displacement did not occur even when traction and compression were released, and the reduced position was maintained. Furthermore, the reduced position was maintained even when the knee joint was passively flexed and extended (flexion 130° and extension 0°) (Figure 4). The left fibula had more than 50% contact and no instability, so fixation was not performed. Postoperatively, the left knee joint was positioned at 0° extension, the left ankle joint at mid‐position, and a Seines splint was applied to cover the area from the mid‐thigh to the toes.

### 2.5. Actual Outcome

**Figure 5:**
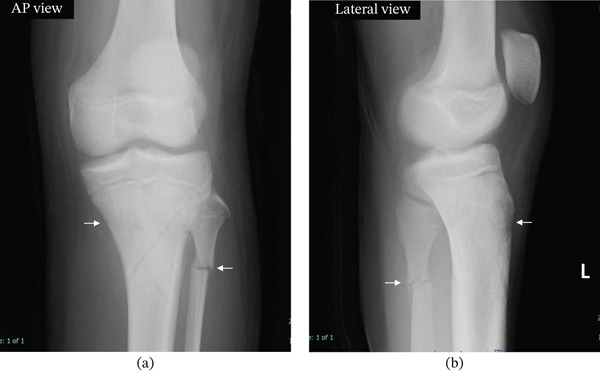
Plain x‐ray images of the left knee at 4 weeks postoperatively. (a, b) Radiographs of the left knee joint at 4 weeks postoperatively. The fracture lines are indicated by arrows. Both the left tibia and fibula show good callus formation with no displacement: (a) AP view and (b) lateral view.

**Figure 6:**
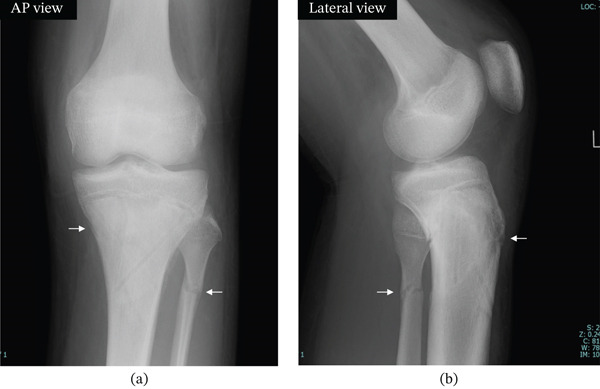
Plain x‐ray images of the left knee at 9 weeks postoperatively. (a, b) Radiographs of the left knee joint at 9 weeks postoperatively. The fracture lines are indicated by arrows. Both the left tibia and left fibula show good bone union, with no signs of displacement: (a) AP view and (b) lateral view.

This case underwent imaging follow‐up until 9 weeks postoperatively, at which point the functional knee brace was discontinued, and full weight‐bearing walking was permitted. Table 2 shows the course of rehabilitation for this case. Furthermore, no apparent adverse events were observed throughout the follow‐up period. Range of motion exercises for the ankle and toes were permitted starting at 2 weeks postoperatively, and range of motion exercises for the knee joint up to 30° of flexion were permitted starting at 3 weeks postoperatively. At 4 weeks postoperatively, evaluation was performed using plain x‐ray images, and since callus formation and bone union tended to be obtained (Figure 5), the K‐wire was removed, the splint fixation was released, and a functional knee brace was applied. Since the wound healing was good, the patient was discharged home at 30 days postoperatively. Subsequently, follow‐up was performed every week with plain x‐ray imaging at the outpatient clinic. At 5 weeks postoperatively, partial weight bearing on the left lower limb (1/3) and knee flexion up to 60° were permitted. At 6 weeks postoperatively, partial weight bearing on the left lower limb (1/2) and knee flexion up to 90° were permitted. At 7 weeks postoperatively, partial weight bearing on the left lower limb (2/3) and knee flexion up to 120° were permitted. At 8 weeks postoperatively, full weight bearing and full flexion were permitted with the functional knee brace in place. At the 9‐week postoperative simple x‐ray evaluation, sufficient bone union was confirmed (Figure 6), and the functional knee brace was removed. Since then, the patient has returned to normal activities, including football, without functional limitations, and there has been no recurrence of fracture or displacement. No deformities such as leg length discrepancy due to growth plate disorders, or genu varum or genu valgum, were observed during the short‐term (9‐week) follow‐up period; however, longer follow‐up (12–18 months, or until skeletal maturity) is necessary to monitor potential growth disturbances.

**Table 2:** Rehabilitation timeline table.

Postoperative week	Immobilization	Range of motion	Weight bearing	Others
0–2	Splint	None	None	
3	Splint	Flexion ≤ 30°	None	
4	Functional knee brace	Flexion ≤ 30°	None	K‐wire removed
5	Functional knee brace	Flexion ≤ 60°	1/3 weight bearing	
6	Functional knee brace	Flexion ≤ 90°	1/2 weight bearing	
7	Functional knee brace	Flexion ≤ 120°	2/3 weight bearing	
8	Functional knee brace	Full	Full weight bearing	
9	None	Full	Full weight bearing	Brace removed

Additionally, regarding vitamin D deficiency, lifestyle guidance, including recommendations for vitamin D intake through diet, was provided to the patient and his parents.

## 3. Discussion

Proximal tibial epiphyseal fractures are relatively rare epiphyseal fractures that primarily occur in young children aged 3–6 years [3]. If not appropriately treated, they may lead to deformities such as leg length discrepancy or valgus deformity, as well as growth disorders [3, 11], necessitating the maintenance of a definitive reduction. Vitamin D deficiency is also suspected as a potential cause in some cases [9]. This case involves a teenager who developed the injury following a minor mechanism of injury, and it is noteworthy for raising concerns about potential bone fragility.

Proximal tibial epiphyseal fractures are often treated with nonsurgical reduction [1]. In cases without displacement, conservative therapy with long‐leg casting for 4–6 weeks is recommended, while surgical therapy using K‐wires, screws, or locking plates is selected for cases with displacement [9, 12]. Moreover, in pediatric fractures involving growth plates, management focused on protecting the growth plate is crucial [13, 14]. When appropriately managed, these injuries are generally associated with favorable outcomes [15].

In this case, surgical intervention was chosen due to the presence of displacement and residual instability following reduction under general anesthesia. Furthermore, as the patient was a pediatric subject with a growth plate, surgical treatment using K‐wire fixation without plates and screws was selected to minimize invasion of the growth plate and the bone. Moreover, fixation using a plate is one option. However, as the proximal tibia is an area with minimal soft tissue, and damage to soft tissue from the plate was anticipated, we judged that fixation using K‐wires would place less burden on the soft tissue in this case. Therefore, we decided on the fixation with K‐wires.

The postoperative course was uneventful, and the patient was able to resume daily activities without significant limitations at 9 weeks postoperatively. Proper reduction and maintenance of the reduced position are crucial. Reports indicate that serum vitamin D levels below 20 ng/mL are associated with an increased risk of fractures [6], and previous studies have reported that approximately 34% of children with a history of fractures have vitamin D deficiency [16], suggesting that vitamin D insufficiency is relatively common in this population. Therefore, vitamin D deficiency may be associated with an increased risk of fractures even in the absence of rickets.

In this case, given the minor mechanism of injury and the unusual presentation of the epiphyseal injury, rickets was considered in the differential diagnosis. However, based on the medical history, imaging findings, and blood test results, the case did not meet the diagnostic criteria for rickets in Japan [17], and asymptomatic vitamin D deficiency was diagnosed. Moreover, there are reports that high vitamin D levels reduce the risk of fractures caused by minor mechanisms of injury [18]. There are case reports [9] of proximal tibial epiphyseal fractures where vitamin D deficiency may have contributed to the development of the fracture, suggesting that vitamin D deficiency may have contributed to the development of the fracture in this case. In cases of a minor mechanism of injury and relatively rare fractures or epiphyseal injuries, rickets and vitamin D deficiency should be considered in the differential diagnosis, and vitamin D levels should be evaluated.

Treatment for rickets or symptomatic vitamin D deficiency involves supplementation with active or natural vitamin D [19], but there is no consensus on intervention for asymptomatic vitamin D deficiency, such as in this case. Since this case was not rickets and did not present symptoms such as tetany due to vitamin D deficiency, we limited our management to explaining the condition to the patient and family and recommending dietary intake. In this case, appropriate bone union and functional improvement were achieved with surgical treatment alone. However, since there is a report that vitamin D deficiency increases the risk of pseudarthrosis [20], it is considered important to evaluate vitamin D levels and consider intervention in pediatric fractures.

## 4. Limitations

While there are reports suggesting that vitamin D deficiency has been reported to be associated with an increased risk of fractures or epiphyseal line injuries [6–8], it cannot necessarily be definitively concluded from a single case such as this that vitamin D deficiency poses a risk of causing epiphyseal line injuries or fractures. In addition, the findings may not be generalizable to all pediatric patients.

## 5. Conclusion

This case report demonstrates that appropriate surgical treatment resulted in a favorable outcome for an unusual proximal tibial epiphyseal fracture in an older pediatric patient. Additionally, due to the minor injury mechanism and the unusual presentation of the epiphyseal injury, we were able to consider rickets and vitamin D deficiency in the differential diagnosis and perform evaluation and intervention accordingly. In pediatric patients with fractures, particularly those with minor mechanisms of injury or unusual fractures/epiphyseal injuries, it is important to evaluate vitamin D levels. Early intervention and nutritional assessment may contribute to favorable clinical outcomes.

## Funding

No funding was received for this manuscript.

## Ethics Statement

Written informed consent for publication of this case report and the accompanying images was obtained from the patient′s parents. The patient was also provided with an age‐appropriate explanation of the study and gave verbal assent. According to institutional policy, single case reports are exempt from formal Institutional Review Board approval.

## Conflicts of Interest

The authors declare no conflicts of interest.

## Data Availability

The data supporting the findings of this study are available from the corresponding author upon reasonable request.
